# A shifting sense of being: A secondary analysis and comparison of two qualitative studies on young-onset dementia

**DOI:** 10.3402/qhw.v9.24756

**Published:** 2014-07-11

**Authors:** Aud Johannessen, Anders Möller, Per K. Haugen, Stian Biong

**Affiliations:** 1 Ageing and Health, Norwegian Centre for Research, Education and Service Development, Vestfold Hospital Trust, Tønsberg, Norway; 2 Ersta Sköndal University College, Gothenburg, Sweden; 3 Faculty of Health Science, Buskerud and Vestfold University College, Drammen, Norway

**Keywords:** Grounded theory, metaphors, Steger, subjective experiences, young-onset dementia

## Abstract

The aim of the present study was to investigate and interpret metaphorical expressions of the lived experiences of everyday life in people with young-onset dementia (YOD) and to compare these findings with findings from an analysis via grounded theory to see if the second analysis adds more knowledge to the topic. In this secondary analysis of data, metaphors from 20 Norwegian men and women living with YOD were investigated. Using Steger's anthropological three-step method, three categories were identified: *Sliding away*, *leaving traces*, and *all alone in the world*. Comprehensively, we understood the metaphors as representing the participants’ *shifting sense of being*. The main findings of the study show that by analysing the data by combining and using both methods, more knowledge to the topic was added. Acknowledging metaphorical expressions as a source of knowledge, this study reflects on how metaphors can be used in therapeutic dialogue. We conclude that metaphors add to the understanding of descriptions of daily life in a more existential way, beyond the results gained from the grounded theory analysis. However, the findings from the analysis via grounded theory included aspects that we did not find when analysing the metaphors.

People who have not reached the age of 65 can be affected by dementia, often referred to as “young-onset dementia” (YOD). Studies show that we need to develop a greater understanding of how life with dementia is experienced by persons with YOD (Bakker, 2013; Johannessen & Möller, 2013; van Vliet, 2012).

No prevalence study has been carried out in Norway, but it has been estimated, based on the work of Harvey, Skelton-Robinson, and Rossor (2003), that up to 1400 young Norwegian citizens are diagnosed with dementia before the age of 65, usually examined within hospitals. The group of persons with YOD is of interest not only because behavioural changes are seen more frequently in this group than in the elderly population with dementia (van Vliet, 2012) but also because the impact on a younger family is great and the costs for society are increasing.

In high-income countries, most people diagnosed with dementia and who are in the early and moderate stages of a dementia disorder live in their own homes (World Health Organization [WHO], 2012). An examination of services that may be offered for patients shows that persons with YOD need specially adapted assistance from the beginning, and as the disease progresses, their need for assistance increases (Bakker, 2013; Beattie, Daker-White, Gilliard, & Means, 2002).

Medical staff need more knowledge so that they contribute to detecting as well as understanding how it is to live with dementia, which, together with psychosocial support, will enable them to provide proper care to persons with YOD. So far, limited research has been performed to lead to the provision of guidelines for such support (Bakker, 2013).

Most people with dementia are capable of expressing their perceived needs (van Vliet, 2012). However, services that have been established for persons with YOD are based on the beliefs of health personnel and are not necessarily in accordance with the wishes of those with YOD (Bakker, 2013; Beattie et al., 2002). For that reason, we carried out a study (Johannessen & Möller, 2013) to increase understanding of the experiences of living with dementia for persons with YOD and to use their narratives to develop services in line with their needs. The original text contained rich descriptions, many of which were metaphorical expressions. After publishing the original findings with grounded theory, we wanted to analyse metaphors in the text, which raised the question of whether such an analysis could add something to the original findings.

In the original study (Johannessen & Möller, 2013), a purposeful sampling strategy was used when recruiting participants (Glaser & Strauss, 1967). This reformulated grounded theory is particularly suitable for the study of people's lives through lived experiences and social interaction. Thus, the transcribed interviews in the original study were analysed in line with the reformulated grounded theory (Corbin & Strauss, 2008). The transcribed verbatim interviews were subjected to three types of coding processes: open, axial, and selective coding. A further description of the grounded theory method used to construct the original findings has been published elsewhere (Johannessen & Möller, 2013).

## Participants

In the original study, four memory clinics in the south of Norway were contacted (Johannessen & Möller, 2013). Health professionals from these hospitals were asked to recruit participants. They performed this task over the telephone or when the persons with YOD had an appointment at their hospital. A total of 22 persons were asked to participate. Two persons declined to participate. Twenty persons with YOD were interviewed (aged 54–67 years; 12 male and 8 female), of whom one was still working part-time. Fifteen of them were married or lived in a cohabiting relationship. Three of the informants had other serious diagnoses. The participants’ diagnosis had been made from 4 months to 3 years prior to the interviews.

## Data collection

The original data were collected during 2010–2011 through individual research interviews (Kvale & Brinkmann, 2010). The interview guide comprised thematic questions focusing on experiences of living with dementia. Depending on answers and reflections, new ideas brought up by the participants raised more questions, and participants were asked to provide additional information. The interviews were carried out in a conversation-based form by the author (AJ), lasted for up to one and a half hours, and were tape-recorded. A professional writer transcribed the interviews shortly after each interview. The author (AJ) performed a quality-control check on the interview transcripts. The interviews were carried out in the participants’ homes, the location they chose, according to their preferences for time and date.

## The original findings (grounded theory)

The category identifications in the original analysis form a model of knowledge telling us what dementia means for the informants in everyday life (Johannessen & Möller, 2013). From analysis of the interviews, two main categories emerged: (1) *the process towards a dementia diagnosis*, and (2) *fighting for dignity*, after being diagnosed with dementia. These two main categories described, from the perspective of persons with dementia, experiences of the process towards a dementia diagnosis, and experiences of fighting for dignity in everyday life when living with dementia, all while knowing that they will reach a point of no return at some point after being diagnosed (i.e., knowing that there will be no recovery). The first core category covers different experiences of life and has two subcategories. The first subcategory describes the informants’ experiences of the *changes* they went through, the early symptoms of dementia, and the subsequent consequences for everyday life. In the second subcategory, these experiences were viewed in parallel with the informants’ feelings about the process of *being diagnosed* and their encounters with the relevant health personnel. The second core category describes the challenges in everyday life and how the informants have tried to maintain their own quality of life after they received the diagnosis. The category encompasses two subcategories describing the informants’ *intrapsychic challenges* and *social challenges*, and it explores the informants’ process and experiences of living with a dementia disorder and the accompanying losses. Apart from the knowledge that there can be no recovery, the informants’ experiences of society's and other's attitudes towards the disorder raised feelings of being stigmatized and lonely. The findings also showed that the informants found great satisfaction in the opportunity to talk about their situation and their own experiences of dementia.

As part of further knowledge development, it could be argued that there is a need to expand the analysis of subjective experiences from this study (Johannessen & Möller, 2013). The aim of the present study was, therefore, to investigate and interpret metaphorical expressions of the experiences of everyday life in people with YOD and to compare these findings with the original findings to determine whether the second analysis added more knowledge to the topic. The research question in the present study was thus formulated: How do persons with YOD describe their everyday life with metaphors, and how might these metaphors be understood?

## Theoretical framework in the present analysis

Lakoff and Johnson's (2003) cognitive-semantic theory of the metaphorical concept claims that metaphors are not a mere ornamental linguistic style, but that they potentially affect our conception of reality. This conception then guides our cognition and actions. Lakoff and Johnson introduce a four-fold typology of metaphors: structural, orienting, ontological, and new metaphors. Each of these contributes in different ways to explain and give meaning to all aspects of individual and cultural processes.

By “structural metaphors,” Lakoff and Johnson (2003) mean that there is no difference between the literal and intended meaning of the utterance. In other words, such metaphors are less dependent on context (i.e., less interpretive). “Orienting metaphors” relate to the notion that human perception is a bodily process that draws from experience within a cultural context. Lakoff and Johnson (2003) claim we both have, and are, our bodies, making us embodied socially (in place) and historically (in time). In contrast to structural metaphors, then, orienting metaphors become more open to interpretation. As orienting metaphors are rooted in bodily and cultural experiences, they often organize whole systems of interlocking meanings. “Ontological metaphors” assist individuals in handling their life experiences by helping them to understand their own experiences, identifying more explicitly the motivations for actions. “New metaphors” are generally structural, but add a dimension of colourful experience to the meaning. The function of colouring is to emphasize, tone down, or hide different aspects. In order to investigate and interpret the experiences of those living with YOD, metaphorical expressions may give us broader and richer descriptions of their experiences.

## Methods

In the present, subsequent study, the experiences of living with dementia are described through an analysis of metaphors. Metaphors allow us to interpret individuals’ understanding of reality and can convey a complex social reality (Ricoeur, 2003). Following Steger's (2007) anthropological three-step method for analysing metaphors, the analysis was first performed by reading the text again and searching for all metaphorical expressions. The process proceeded with the identification and selection of metaphors specifically representing everyday life. These were then analysed for their similarities and differences, forming the categories presented in the findings. Finally, we returned to the original text and investigated the implications of the metaphors in their particular context, with the meaning assigned to them by the participants.

### Ethics

This study follows the ethical principles outlined in the revised Helsinki declaration (World Medical Association [WMA], 2008) and was approved by the Regional Committee for Ethics in Medical Research, Southern Norway. Person-identifiable data have been deleted from all stored transcripts to provide confidentiality. The participants were required to sign a written form of consent and were informed that participation or non-participation in the project would not in any way influence their follow-up or treatment. The researcher had no influence on the follow-up or treatment of the participants. Before the interview took place, the possibility of immediate intervention was provided should the interview cause negative psychological reactions, but negative reactions were not observed.

## Findings

The first part of this section presents the findings from the analysis of metaphors, and the second part presents a comparison between the original analyses and the metaphors.

### The first part (analysis of metaphors)

From the initial readings in step one, it became evident that the shock of the discovery and the struggle to cope with the diagnosis were central to participants’ experiences of living with dementia. Participants’ stories shared sentiments of feeling that their thoughts were just sliding away or were stopped up. A feeling of being outside themselves and a growing feeling of being outside society were expressed through orienting metaphors. These metaphors contained the feelings of stigma linked to the disorder. As time passed after the diagnosis, the metaphors used by patients expressed ways of coping with the situation. From this position, existential reflections expressed by ontological metaphors were used. Reflections on how the disorder would develop and end were also expressed. From all the identified metaphors reflecting on the participants’ everyday life, we were able to abstract three different categories: *sliding away*, *leaving traces*, *and all alone in the world*.

### Sliding away

The constructed meaning of *sliding away* concerns bodily and social aspects of the illness. The symptoms were linked to the cognitive experience of them, and this experience was expressed through orienting metaphorical expressions about everyday life. Often these experiences gave rise to a shifting notion of being present and sliding away at the same time:

Suddenly, I am completely gone. It sometimes happens that I suddenly wake up and then slide out of it again; my thoughts somehow fall out, actually. It has started to slip a little bit out with me, and I feel completely outside sometimes.

These cognitive changes were understood and metaphorically expressed as a loss of natural functions. These shifts and changes could give rise to feelings of being outside oneself, but also feelings expressed like, “I am actually very healthy except for what is in my head …”

Socially, the participants commented that other people believed that they would be completely “gone” someday. Linked to the shifting memory losses, experiences of stigma could arise:

Even if I knew that there was something wrong, and I knew that it was probably Alzheimer's disease, it is still hard receiving it written down, as though I am being tamped in my forehead. My thoughts can be bottled up sometimes, and my thoughts just slide away, but I know that it comes back.

### Leaving traces

The constructed meaning of *leaving traces* concerns how the participants experienced stigma. Stigma linked to the disorder led initially to anger. Anger was also linked to falling out of work and to receiving a diagnosis. After a while, life improved, but it was still difficult because of the lack of knowledge of what was to come. Coping with stigma was described as a struggle to find a way back to activities and back to a social life, and the benefit of medication in this process was noted. The metaphors became more ontological, describing how the participants constructed strategies for everyday life so as to not forget or be forgotten:

I am careful to leave traces of what I have done, so that I remember what I have done.

Managing life with the disorder and its stigma was metaphorically expressed as an attempt to continue with ordinary activities and, because of memory loss, how training the brain was important to in the process of leaving traces:

You have got dementia . . . and . . . that in itself is not so bad, so far so good, but you may become an idiot. I have found out that if you do not use your brain, then you go straight down.

### All alone in the world

The constructed meaning of *all alone in the world* concerns how the participants create meaning in their everyday life. In different ways, life went on for the participants after their diagnoses. Ontological metaphors expressed existential aspects of being alone and together at the same time:

You believe that you are all alone in the world in this respect, and it is quite good to hear that there are others who are likely to be experiencing [the disease] in the same way.

Expressing the feeling of being all alone, one participant questioned receiving the diagnosis and having no one to talk with. The participants commented that they did not worry all that much about what was to come. They described being alone with their thoughts about how the disorder would progress and noted that this experience probably went differently for everyone. Metaphorically, they expressed ways of coping with the fear of what might come:

I am not there yet, so we just have to hope that it stops.

The main finding was the description of what the three categories had in common. In this process, we comprehensively interpreted participants’ metaphors of their everyday life as a whole as representing their “*shifting sense of being*.” To understand the bodily symptoms of the disorder and the accompanying social changes as a part of the disorder allowed them, after some time, to be able to build up coping strategies for themselves. Both emotional and problem-solving coping strategies were useful to them as they continued with life after the diagnosis. In noting the struggle for understanding and coping, they described by ontological metaphors how stigma was linked to the disorder. Challenging existential thoughts were balanced by a commitment to taking one day at a time and an acceptance that their life was as good as it could be.

### The second part (the comparison between the two analyses)

The core categories in the first of these two analyses were *towards a dementia diagnosis* and *fighting for dignity*. The core category in the analysis of metaphors was *a shifting sense of being*. The grounded theory analysis incorporated from Corbin and Strauss (2008) was useful in exploring the participants’ experiences of what happened, of what they have, and of their social experiences. Stegers’ (2007) anthropological three-step method for analysing metaphors highlights aspects of being and becoming, that is, existential but also bodily and social aspects of living with YOD.

The findings from the analysis via grounded theory included aspects that we did not find in the metaphorical expressions, such as participants’ experiences with health personnel and their satisfaction with opportunities to talk about their situation. The comparison of the two findings did show congruencies of different findings as well, such as participants’ experiences of the symptoms of the disorder, their struggle with everyday life after being diagnosed, and their coping strategies. The stigma linked to the disorder and the feeling of being outside society was also found in both analyses.

## Discussion

The main findings show that by analysing the data with both of the aforementioned methods and subsequently combining these analyses, substantial knowledge was contributed to the topic. In the second analysis as a whole, the metaphors expressing experiences of everyday life could be understood as *a shifting sense of being*, thereby adding existential aspects to the first analysis. By combining the findings from the two kinds of analyses, the complex lives of persons with YOD can further be illuminated. To contribute to a broader perception of the complex situation of persons with YOD, the findings will be discussed on an *individual level*, on a *group level*, and on a *societal level*.

On an *individual* level, the process of understanding the bodily symptoms of the disorder, and the accompanying social changes as a part of the disorder, allowed the participants, after some time, to establish coping strategies, which were expressed throughout orienting metaphors. To help people living with YOD on an individual level with their processing of experiences and demands, health professionals should develop careful listening skills and should be trained to understand the value of what they hear. According to Borg (2007), it is essential to listen to the experiences of users, for it is in this context that persons with YOD try to create meaning in their lives. Meaning in life is shown to be a vital part of recovery in mental health problems (Deegan, 1988). Bakker (2013) has shown that persons with YOD risk developing a poor quality of life as a consequence of the illness. Considering this, it is important to focus on dialogue and on arenas that can help persons with YOD to create meaning in their lives in spite of cognitive impairments, to help maintain their physical and mental health, and to promote social well-being.

Furthermore, the participants seemed to ground their preferred metaphorical construction in cultural ideals of an active self when communicating challenging existential thoughts and expressing their fight to maintain dignity. These ideals moved them to continue with social activities and to take one day at a time. This finding is in line with Antonovsky's (1987) perspective on general resistance resources as a part of coping with stressors. Antonovsky deems social support to be one of the most important aspects of these resources. In order to stimulate these resources for coping with stressors on an individual level, metaphorical expressions could act as a gatekeeper in the dialogue with persons with dementia and contribute to developing services.

On a *group* level, building a philosophy of dementia care by listening to patients’ perspectives and stories is important. According to Martinsen (1990), understanding the situation of others is a prerequisite to being able to act caringly. In this context, being able to listen to and act on others’ language, including their use of metaphors, can make a difference. By listening carefully, the time from the onset of the illness to the diagnosis might be compressed (van Vliet, 2012). To contribute to helping persons with YOD, it is important to listen to linguistic expressions, an aspect of care that has often been omitted (Bakker, 2013). Linguistic and semiotic aspects of language and metaphor can express the ways in which different stressors are perceived. Metaphors can then be said to have a role in the development of a therapeutic alliance. Thus, by attending to and addressing metaphorical expressions of people with dementia, health professionals can signal acceptance and recognition of subjectivity in order to come closer to their needs (Skårderud, Haugsgjerd, & Stänicke, 2009).

On a *societal* level, it is important that health professionals support persons with YOD in their efforts to live an active life. The Ottawa Charter (WHO, 1986) highlights the importance of creating supportive environments. Both problem-solving and other coping strategies were useful to the participants in keeping up with activities. In the struggle for understanding and coping, participants described by ontological metaphors how stigma was linked to the disorder. The WHO (2012) emphasizes that people with dementia and their caretakers often have unique insights into their condition and lives. To remove stigma and to contribute to a better life for persons with YOD and their families, their metaphorical language and insights should be used to validate and develop services that relate to them.

The findings show that existential metaphors of feelings of being alone were expressed in both analyses. This shows how important it is to listen to subjective expressions, because such a diagnosis has serious consequences for those who are affected and results in a significant loss of a healthy quality of life. To be a person living with YOD means that you are likely to suffer from a loss of social roles, such as being the financial provider or being a spouse or a parent, resulting in a loss of personal identity (Millenaar et al., 2013; van Vliet, 2012). As a consequence of the experiences with symptoms like those that participants described in the category sliding away, people with YOD will often in an early stage of the disease become unemployed (Bakker, 2013; Millenaar et al., 2013). The symptoms of dementia, the feeling of stigma linked to the disorder, and the feeling of being all alone contribute to the reality that people with YOD may lose contact with society and may lose their ability to give meaning to life. Family caretakers and adult children are at risk of developing stress-related health problems and experience more relational difficulties and family conflicts, which are caused by the burden of care and symptoms related to dementia (Bakker, 2013; Barca, Thorsen, Engedal, Haugen, & Johannessen, 2014; Millenaar et al., 2013).

The symptoms of dementia are often mistaken for other more common illnesses, such as depression and anxiety. The diagnostic process is therefore longer than it is among the elderly, and causes more negative experiences and a greater burden regarding the diagnostic process for these families (Barca et al., 2014; van Vliet, 2012). It is therefore important to increase awareness among experts, so that they listen more carefully to people with depression, anxiety, and cognitive impairment who have not reached the age of 65 and organize hospital departments and municipality services so that the period before receiving a diagnosis can be compressed in order to reduce or prevent stress-related health problems (Bakker, 2013; Barca et al., 2014; Johannessen & Möller, 2013).

The organization of services and meeting places, as Bakker (2013) points out, is also important to reduce feelings of being alone for those with YOD, or to give them a possibility to maintain participation in society and contribute to their quality of life, in line with Buber's (1923) illustration of how humans find meaning in life in relationships, when they receive confirmation through others. Because of the low prevalence of YOD, this is not always possible in a single-municipal community (Harvey et al., 2003). In this situation, services must be offered to help persons with YOD to cope with their feelings of being alone and of isolation and to help them find other solutions to build meaning into their lives through relationship, in line with Buber's (1923) illustrations.

The metaphor-based approach adopted here contributes a deeper understanding of the situation for persons with YOD. If we had been more aware of these metaphors when collecting the data in the original study, other follow-up questions could have been asked to provide richer data.

## Conclusion

We conclude that metaphors add a deeper level of meaning to the descriptions of everyday life provided by people with YOD, introducing an existential element that reaches beyond the results gained in the grounded theory study. An individual's perception of everyday life can be expressed through metaphors. Health professionals should therefore work to listen carefully and to truly understand the value of what they hear. Metaphorical language may in that way provide a target method to validate and develop services. Findings from the grounded theory analysis included aspects that we did not find when analysing for metaphors. In addition, this study underlines that persons with YOD have a feeling of being outside society, which can be explained by the stigma linked to the disorder, and by the disorder itself. People with YOD also have a feeling of being outside themselves, but they are still able to build up coping strategies.
